# HEMATOHIDROSIS – A RARE CLINICAL PHENOMENON

**DOI:** 10.4103/0019-5154.55645

**Published:** 2009

**Authors:** H. R. Jerajani, Bhagyashri Jaju, M. M. Phiske, Nitin Lade

**Affiliations:** *From the Department of Dermatology, Lokmanya Tilak Municipal Medical College and General Hospital, Sion, Mumbai – 400022, India*

**Keywords:** *Hematohidrosis*, *blood*, *sweat*, *heperhidrosis*

## Abstract

Hematohidrosis is a very rare condition of sweating blood. A case of hematohidrosis is reported. There are only few reports in the literature.

## Introduction

Hematohidrosis is a rare condition in which a human being sweats blood.[1] Leonardo Da Vinci described a soldier who sweated blood before battle. Jesus Christ experienced hematohidrosis while praying in the garden of Gethsemane before his crucification as mentioned in the Defenders Bible by Physician Luke as “and being in anguish he prayed more earnestly and his sweat was like drops of blood falling to the ground.”

The causes of hematohidrosis have been divided into nonreligious and religious. The nonreligious causes are as a component of systemic disease, vicarious menstruation (bleeding from a surface other than the mucous membrane of the uterine cavity that occurs at the time when normal menstruation should take place), excessive exertion, psychogenic, and unknown factors. Duan *et al.* reported hematohidrosis associated with primary thrombocytopenic purpura.[2] Migliorini described a case of hematidrohosis otorrhea with otoerythrosis.[3] Dubeikovskaia reported hematohidrosis in a 8-year-old child.[4]

The religious cause is a stigma, which formerly meant a spot, a sign, a wound, or a mark branded on a slave. From the time of Christ's crucifixion, this term took on the special meaning as the reproduction of the wounds on palms, soles and crown that Christ suffered on the cross and it was believed to be supernaturally imposed by God. Jacobi (1923), quoted by Klauder, reported 300 instances of stigma (stigmata). Most of the stigmata patients were females both Catholics and non Catholics.

## Case Report

A 72-year-old male consulted us for staining of undergarments with blood, in the area confined to the abdomen for 2 months, especially in the morning. He has suffered from continuous mental stress for two years due to family feud. There was no history of trauma to abdomen or genitals, bleeding disorder, excessive consumption of coloured diet or allergy to food or drugs. He was a vegetarian and did not come in contact with meat and poultry products. He did not report any blood-stained discharge from the urethra and anal region. He denied history of extramarital sexual contact or development of STI.

**Figure 1:**
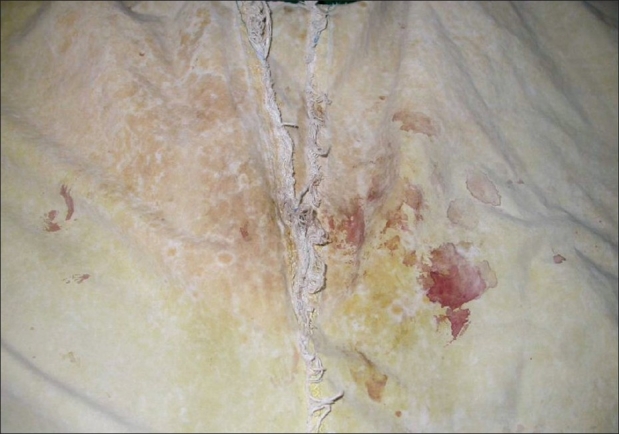
Blood stains visualized on undergarments covering anterior part of abdomen

**Figure 2:**
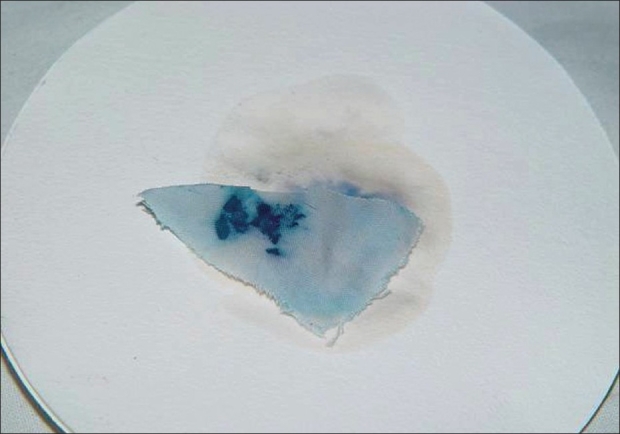
Positive Benzidine test

Cutaneous examination revealed trichomycosis axillaries and yellow staining of clothing, which were in contact with axillae and chest wall suggestive of chromhidrosis. Blood stains were not seen on the skin surface. They were visualized only on the portion of undergarment covering anterior part of abdomen [Figure 1], but not the genitals, perianal area, and buttocks. Routine hemogram and biochemical investigations to look for any systemic abnormality were within normal limits. Urine microscopy and urethral swab revealed no abnormality. Benzidine test to detect blood pigment on the undergarments [Figure 2] was positive. Hemochromogen test for confirming the blood pigment to be human blood pigment could not be performed due to nonavailability. Biopsy done during remission revealed an unremarkable epidermis, capillarysized vessels with RBCs in their lumen in the dermis along with papillary dermal oedema and dermal melanophages. Special stains to detect hemosiderin (percian blue) was positive. Psychiatric evaluation detected depressive disorder. On the basis of his clinical presentation, presence of depressive disorder and a positive benzidine test, diagnosis of hematohidrosis was made. Apart from regular counselling for his depressive disorder, he received no other systemic therapy.

There was complete subsidence of bleeding after 15 days, with no reports of similar complaints at follow up in 6 months and one and half year.

## Discussion

Hematohidrosis also known as Hematidrosis, hemidrosis and hematidrosis, is a condition in which capillary blood vessels that feed the sweat glands rupture, causing them to exude blood, occurring under conditions of extreme physical or emotional stress.[1] Manonukul *et al*. proposed the term “hematofolliculohidrosis” because it appeared along with sweat-like fluid and the blood exuded via the follicular canals.[5]

Various causative factors have been suggested by Holoubek, like component of systemic disease, vicarious menstruation, excessive exertion, psychogenic, psychogenic purpura, and unknown cause.[5]

Acute fear and intense mental contemplation are the most frequent causes, as reported in six cases in men condemned to execution, a case occurring during the London blitz, a case involving fear of being raped, a case of fear of a storm while sailing, etc.[6] In our case, the probable cause for hematohidrosis was chronic stress, as the other causes were ruled out by detailed investigations. Hysterical mechanisms and psychosomatic disorders are also believed to induce bleeding.[6] Psychogenic purpura is supposed to be caused by hypersensitivity to the patients' own blood or autoerythrocyte sensitization and is characterized by repeated crops of ecchymoses, gastrointestinal bleedings, and hematuria.

Another type of bleeding through skin is psychogenic stigmata; a term used to signify areas of scars, open wounds or bleeding through the unbroken skin. Patients belonging to this group were found to be frequently neurotic.The clinical findings of this type are a slight elevation of skin before prolonged oozing of blood, a peasized bluish discoloration on patient's palm and erysipelas-like lesion. Copeland reported a patient who developed bleedings from her old scars whenever she had serious anxiety.[6]

The etiopathogenesis according to Dr. Frederick Zugibe is that multiple blood vessels which are present in a net-like form around the sweat gland constrict under pressure of stress. As the anxiety increases, the blood vessels dilate to the point of rupture. The blood goes into the sweat glands, which push it along with sweat to the surface, presenting as droplets of blood mixed with sweat. The extravasated blood has identical cell components as that of peripheral blood. The severe mental anxiety activates the sympathetic nervous system to invoke the stress-fight or flight reaction to such a degree as to cause hemorrhage of the vessels supplying the sweat glands into the ducts of the sweat glands. Effect on the body is weakness and mild to moderate dehydration from the severe anxiety and both blood and sweat loss.[7] Manonukul *et al*. has recently proposed that there may be some defects in the dermis causing stromal weakness. These defects will communicate with vascular spaces in the dermis and they will eventually dilate and enlarge as blood-filled spaces when the blood comes in. After that, they will exude the blood out by either via follicular canals or directly on to the skin surface and this will occur whenever the positive pressure inside is enough. Afterwards, they will collapse leaving no scar. This phenomenon acts like a balloon, waxes and wanes and thus explains why these bleedings are sometimes intermittent and self-limiting. Immediate biopsy is important because a late biopsy, after these spaces collapse, will not help in identifying them.[6] Skin pathohistological study by Zhang *et al*. revealed some intradermal bleeding and emphraxised (obstructed) capillaries. No abnormality was found in sweat glands, hair follicles, and sebaceous glands. They concluded that pathological basis for hematohidrosis might be a distinctive vasculitis.[7]

Biopsy in our patient done during remission did not reveal any blood filled vascular spaces, intradermal bleeding, obstructed capillaries or abnormality in hair follicle, sebaceous or sweat glands.

Diagnosis of hematohidrosis is by Benzidine test in which hemoglobin in blood reacts with hydrogen peroxide liberating oxygen, which then reacts with organic reagent producing a green to blue coloured compound. Hemochromogen test confirms that the blood is of human origin. In this test, pyridine causes reduction of hamoglobin resulting in characteristic salmon-pink crystals of pyridine hemoglobin observable under microscope.

Unique features of our case include localised involvement of the abdominal area, hitherto unreported. Excellent recovery on psychiatric counselling highlights the relationship between psychogenic causes and hematohidrosis.
